# Proteomic Profiling and Pathway Analysis of Acid Stress-Induced Vasorelaxation of Mesenteric Arteries In Vitro

**DOI:** 10.3390/genes13050801

**Published:** 2022-04-29

**Authors:** Ipsita Mohanty, Sudeshna Banerjee, Arabinda Mahanty, Sasmita Mohanty, Nihar Ranjan Nayak, Subas Chandra Parija, Bimal Prasanna Mohanty

**Affiliations:** 1 ICAR-Central Inland Fisheries Research Institute, Biochemistry Laboratory, Proteomics Unit, Barrackpore, Kolkata 700120, India; mohantyi@chop.edu (I.M.); sudeshnabanerjee1986@gmail.com (S.B.); mahantyarabinda1@gmail.com (A.M.); 2 Department of Pharmacology and Toxicology, College of Veterinary Sciences and Animal Husbandry, Orissa University of Agriculture and Technology, Bhubaneswar 751003, India; profscparijaouat4691@gmail.com; 3 Departments of Pediatrics, Children’s Hospital of Philadelphia Research Institute, The Raymond and Ruth Perelman School of Medicine, University of Pennsylvania, Philadelphia, PA 19104, USA; 4 ICAR-National Rice Research Institute, Cuttack 753006, India; 5 Department of Biotechnology, Faculty of Science & Technology, Rama Devi Women’s University, Bhubaneswar 751022, India; sasmita.mohanty@rdwu.ac.in; 6 Department of Obstetrics and Gynecology, UMKC School of Medicine, Kansas City, MO 64108, USA; 7 Indian Council of Agricultural Research (ICAR), ICAR-Fisheries Science Division, Room No. 308, Krishi Anusandhan Bhawan II, New Delhi 110012, India

**Keywords:** acid stress, actin regulation, ATP synthetase β, mesenteric artery, metabolic acidosis, proteome, vascular tone, vasorelaxation

## Abstract

Although metabolic acidosis is associated with numerous pathophysiological conditions and its vasorelaxation effects have been well described in different animal and culture models, the molecular mechanisms of acidosis-induced vasorelaxation are not fully understood. Mesenteric artery models have been used extensively to examine the vascular response to various pathophysiological conditions. Our previous studies and several other reports have suggested the vascular responses of goat mesenteric arteries and human arteries to various stimuli, including acidic stress, are highly similar. In this study, to further identify the signaling molecules responsible for altered vasoreactivity in response to acidic pH, we examined the proteomic profile of acid stress-induced vasorelaxation using a goat mesenteric artery model. The vascular proteomes under acidic pH were compared using 2D-GE with 7 cm IPG strips and mini gels, LC-MS/MS, and MALDI TOF MS. The unique proteins identified by mass spectroscopy were actin, transgelin, WD repeat-containing protein 1, desmin, tropomyosin, ATP synthase β, Hsp27, aldehyde dehydrogenase, pyruvate kinase, and vitamin K epoxide reductase complex subunit 1-like protein. Out of five protein spots identified as actin, three were upregulated > 2-fold. ATP synthase β was also upregulated (2.14-fold) under acid stress. Other actin-associated proteins upregulated were transgelin, desmin, and WD repeat-containing protein 1. Isometric contraction studies revealed that both receptor-mediated (histamine) and non-receptor-mediated (KCl) vasocontraction were attenuated, whereas acetylcholine-induced vasorelaxation was augmented under acidosis. Overall, the altered vasoreactivity under acidosis observed in the functional studies could possibly be attributed to the increase in expression of actin and ATP synthase β.

## 1. Introduction

Metabolic acidosis is a relatively common clinical condition, characterized by a decrease in blood pH and bicarbonate concentration, affecting a number of organ systems in the body and often progressing to neurological and cardiac complications [1,2]. Acute metabolic acidosis affects the cardiovascular system, leading to decreased cardiac output, impaired oxygen delivery, arterial dilatation, decreased ATP production, and predisposition to arrhythmias [3,4,5]. In certain surgical interventions, prolonged occlusion of the arteries results in accumulation of metabolic waste and lactate in the ischemic tissues. Reperfusion through vascular declamping for recovery often advances through ischemic tissue acidosis, progressing to low blood pressure and hypovolemic shock, followed by impaired vascular permeability and vascular dysfunction [3,4,5]. Similarly, acute mesenteric ischemia is an emergency condition of humans that requires prompt diagnosis and instant medical intervention [6]. The first few hours of ischemia are critical for the retrieval of the affected bowel segment and the return to productive status post recovery. Thus, identification of its clinical prognostic/diagnostic biomarkers is important for reducing mortality and morbidity of humans and animals [6]. Hence, there is an urgent need for aggressive correction of acidosis and management of systemic hypotension and circulatory shock. The present study fills in an important gap regarding knowledge of signaling molecules responsible for vascular pathophysiological disturbances in acidic stress.

Acidosis often leads to devastating consequences, such as ischemia, hypoxia, metabolic disorders, vascular shock, and hypotension. Therefore, predicting the risk of acidosis and designing new prophylactic strategies are serious challenges of modern medicine. Mesenteric arteries are systemic, resistance-sized vessels that play an important role in the control of blood pressure and contribute towards the regulation of peripheral vascular resistance [7]. It is expected that any change in the normal physiology of dilatation due to acidosis of mesenteric arteries can affect health. The superior mesenteric artery (SMA) supplies blood to a major part of the gastrointestinal tract extending from the lower part of the duodenum to the transverse colon and pancreas. The mesenteric arterial bed is complex, characterized by multiple redundant interconnecting branches, allowing for a rich blood supply to a major portion of the splanchnic vascular bed that extends from the whole of the small intestine up to the middle third of the transverse colon [8]. Thus, mesenteric vessels play an important role in maintenance of vascular tone under physiological conditions. The present study aims at identifying the signaling molecules involved in altered vasoreactivity under acidic pH using the superior mesenteric artery (SMA). Goat mesenteric arteries are used here as their anatomy, diameters [9], and vascular physiology [10] are similar to those of human mesenteric arteries. The results of this study have implications for understanding the pathophysiology and rational therapeutic targeting of acidosis-associated vascular disorders.

Proteomics comprises the large-scale, systematic study of protein structure and function, usually with respect to a defined entity: a pathway, organelle, cell, tissue, or organism. Clinical proteomics, in which mass spectrometry (MS)-based proteomic approaches are used, involve the detailing and quantification of proteomes of normal or diseased tissues to identify peptides, proteins, and post-translational modifications that support early disease detection, facilitate diagnosis, inform prognosis, guide therapy, monitor disease activity, or identify new therapeutic targets [11]. Proteomics represents the current frontier technology and, of late, has been used to study vascular diseases [11,12].

Proteomic analysis of whole-cell and tissue extracts by means of two-dimensional electrophoretic separation (2D-GE) coupled to mass spectrometric analysis is a powerful approach for the identification of protein signatures of pathogenetic, diagnostic, and/or prognostic significance. It is an expanding field of investigation which, in combination with other “omics”, may enhance understanding and/or identify biomarkers of vascular pathophysiology [13]. In vascular research, especially in veterinary research, proteomics is in its infancy, and proteomics findings will form the basis for future investigations exploring the role of novel proteins in the vasculature [13]. Any dysfunction in vascular permeability is observed as inflammation, tumor metastasis, angiogenesis, and atherosclerosis, and such processes can be identified if proteome maps from vessel proteins under normal physiology and pathophysiology are compared. Recently, the study of whole-cell transcriptomes and proteomes has allowed lymphatic system researchers to characterize diverse endothelial cell types at the molecular level and perform comparative studies [14,15]. A previous study of proteomics profiling of rat renal proximal convoluted tubules following metabolic acidosis identified the primary pathways altered during the onset of metabolic acidosis [2]. Moreover, proteomic profiling has also been performed using apical membrane isolated from proximal convoluted tubules to characterize the physiological response to the onset of metabolic acidosis [16]. The present study was performed on superior mesenteric arteries (SMAs) of goats to understand the mechanism behind altered vasoreactivity under acid stress. In the present study, we used the mesenteric artery due to their sensitivity to fall in extracellular pH (pH_o_) and obtaining reproducible results using isolated arterial rings [17,18]. To the best of our knowledge, it is the first such study on whole goat mesenteric arteries. Using an ex vivo approach, arterial rings were incubated in neutral (pH_o_ 7.4) and acidic pH (pH_o_ 6.0 and 6.8) for 3 h [18,19,20] to be consistent with the duration of functional studies and used for proteomic analysis. Considering the high sensitivity of mesenteric arteries to low pH and our previous studies showing increased vasorelaxation under mildly acidic conditions (pH_o_ 6.8) [18], in this study, we have extended our approach to investigate the effects of severe acidic pH_o_ (6.0) on the vascular contractile machinery of SMA. Then, we investigated the proteomic changes in the mesenteric artery using 2D-GE, liquid chromatography with tandem mass spectrometry (LC-MS/MS), and matrix-assisted laser desorption/ionization time-of-flight mass spectrometry (MALDI TOF/MS) to identify the proteins or signaling molecules that could be responsible for the observed pharmacological events. Our results provide new insights into a possible adaptive mechanism for maintenance of vascular tone under acute acid stress through upregulation of actin proteoforms, actin-associated proteins, and mitochondrial ATP synthase and suggest that extracellular acidosis likely affects the contractile machinery of SMA by regulating the dynamic organization of actin cytoskeleton.

## 2. Materials and Methods

### 2.1. Animals

This project has been reviewed and approved by the Institutional Animal Ethical Committee (registration no: 433/CPCSEA/20/06/2001) vide ID130/CVS/dt.31.03.2015. Mesenteric arteries from *Capra hircus* (Black Bengal goats, 20–25 kg body weight, both male and female, age 13–15 months) were used as the experimental material.

### 2.2. Collection of Mesenteric Artery

A piece of omentum with SMA supplying the small intestine, especially duodenum and jejunum, was collected in chilled modified Krebs–Henseleit solution (MKHS) from freshly sacrificed adult goats from the slaughterhouse in Bhubaneswar. Following careful examination of common mesentery, a portion of the SMA just before its bifurcation into secondary or tertiary branches was dissected out and placed immediately into aerated ice-cold MKHS and maintained at pH_o_ 7.4.

### 2.3. Preparation of Superior Mesenteric Artery and Tension Recording

SMA rings of 1.5–2 mm were isolated, cleared of fat and adventitious tissue, and mounted between two stainless steel triangular hooks in an automatic organ bath (Pan Lab) containing MKHS adjusted to pH_o_ 7.4, 6.8, or 6.0 using 1N HCl [18,19]. The arterial rings were equilibrated for 1.5 h under a resting tension of 1.5 g in 20 mL of MKHS (37.0 ± 0.5 °C) with continuous aeration with carbogen (95% O_2_ + 5% CO_2_). During this period, the bathing fluid was changed every 15 min. The isometric contraction/dilation tension was measured by a highly sensitive isometric force transducer (Model MLT0201, AD instrument, Bella Vista, Australia) and calculated using Chart 7.1.3 software.

### 2.4. Isometric Contraction Study

#### 2.4.1. Histamine- and KCl-Induced Concentration-Related Contractile Responses at pH_o_ 7.4, 6.8, and 6.0

After equilibrating the arterial rings in MKHS (pH_o_ 7.4, 6.8, or 6.0) for 45 min, histamine (1 nM–100 µM) or KCl (20–80 mM) induced contraction was elicited by successive addition to the bath in increments of 1 log unit at 4 min intervals. Net tension (g) under plateau at each concentration was recorded and plotted against –Log (M) concentration of histamine/KCl to elicit a sigmoid concentration response curve (CRC) for comparison. The percent maximal response (E_max_) and –LogEC_50_/pD_2_ were calculated for SMA rings under different pH_o_ ranges and compared.

#### 2.4.2. NA- and KCl-Induced Sustained Contraction Relaxed with Ach and SNP

Following a 90 min equilibration, submaximal concentrations of NA (10 μM) or KCl (60 mM) were added to the baths to obtain an initial phasic followed by a sustained contractile response. Ach or SNP (1 nM–100 μM) was then added in increments of 1 log unit every 4 min to NA (10 μM) precontracted SMA rings. The net tension (g) at each concentration of Ach or SNP was calculated. The percent contractile response at each concentration was calculated by taking the net plateau tension (g) induced by NA as 100%. The percent relaxation response (E_max_) at each concentration was obtained by reducing the percent contractile response from 100%.

### 2.5. Preparation of Protein Extracts and Quantification

SMA rings were incubated in MKHS (pH_o_ 7.4, 6.8, or 6.0) for 3 h under continuous aeration to simulate the exposure time in organ baths, and then employed for proteomic analysis. To simulate the washes in organ baths, the MKHS that was used for incubation was replaced every 15 min with fresh MKHS, pH-adjusted. Protein extracts from SMA walls were prepared following a published protocol [21]. Briefly, tissue samples were homogenized in rehydration buffer (6 M urea, 2% CHAPS, 50 mM DTT, 0.2% Bio-Lyte 5/10 ampholyte, and 0.001% bromophenol blue). The homogenates were centrifuged at 11,500× *g* at 4 °C for 15 min. The protein content of the supernatants was determined by Bradford protein assay, using BSA as a standard [22]. To verify equal loading for 2D-GE, the samples were initially analyzed by SDS-PAGE by loading equal quantities of protein (control vs. experimental), as measured by Bradford assay, and visually assessed after staining of the gels [23].

### 2.6. Gel Electrophoresis

Soluble protein extracts were separated by 2D PAGE following a standard protocol [24]. Briefly, immobilized pH gradient (IPG) strips (7 cm, pH 5–8, Bio-Rad) were used for isoelectric focusing. Protein samples (150 µg) were premixed with “rehydration buffer” (6 M urea, 2 M thiourea, 2% CHAPS, 50 mM DTT, 0.2% Bio-Lyte 5/8 ampholyte, and 0.001% bromophenol blue), and rehydration of the IPG strips was carried out for 12 h. Isoelectric focusing (IEF) was performed at a current of 50 µA/strip at the stated voltage gradient: 100 V for 10 min, 250 V for 20 min, 4000 V for 2 h, and 4000 V for 10,000 Vh. After isoelectric focusing (IEF), the focused strips were equilibrated with “equilibration buffers” I (reducing buffer, 0.375 M tris-HCl, pH 8.8, 6 M urea, 20% *v*/*v* glycerol, 2% SDS, and 130 mM DTT) and II (alkylating buffer, 0.375 M Tris-HCl, pH 8.8, 6 M urea, 20% *v*/*v* glycerol, 2% SDS, 135 mM iodoacetamide) for 15 min each, and then placed on SDS polyacrylamide slab gels for second dimension electrophoresis. The second dimension SDS-PAGE was performed using a 12% separating gel with 5% (*w*/*v*) stacking gel (thickness 1 mm). The gels were stained with Coomassie brilliant blue. Molecular weight markers (Sigma-Aldrich Chemicals Private Limited, St. Louis, MO, USA, #S8445, molecular weight range 6500 to 200,000 Da) were used for calibration.

### 2.7. Gel Image Analysis

Stained gels were scanned, and the images were acquired by Image Scanner III LabScan 6.0 (GE HealthCare, Chicago, IL, USA). The 2D gel image analysis software PDQuest (Bio-Rad, Hercules, CA, USA) version 7.2.0 was used for gel-to-gel matching and identifying differences in the proteomes. Each of the three pH-specific sample sets was analyzed using three independent biological replicates on three different 2D gels. The gel images were normalized in the PD Quest software to even out differences in staining intensities between gels. Each matched protein spot was assigned a unique sample spot protein (SSP) number in the PD Quest software. For gel comparison, a statistical approach was applied when determining differentially expressed proteins using the PD Quest software. The Student’s *t*-test was performed using a 95% significance level to determine which proteins were differentially abundant between experimental and controls tissues. The differentially abundant proteins were identified by MALDI TOF/MS and LC-MS/MS.

### 2.8. MALDI TOF/MS and LC-MS/MS

Protein spots of interest were cut from the 2D gels, destained in methanol and ammonium bicarbonate buffer, and digested overnight with trypsin. Selected protein spots were analyzed by LC-MS/MS following standard techniques, described earlier [25]. A few protein spots were also analyzed by MALDI TOF mass spectrometry using a 5800 Proteomics Analyzer (AB Sciex, Massachusetts, United States) [26]. For protein identification, peptide masses from trypsin digests derived using the MALDI TOF/MS were used to search against the Ludwig NR Database with taxonomy set to Mammalia using the Mascot sequence matching software (Matrix Science, Chicago, IL, USA, www.matrixscience.com (accessed on 18 April 2020)). The MASCOT search parameters were as follows: peptide mass accuracy was 100 ppm, and protein modifications allowed included cysteine as the *S*-carbamidomethyl derivative and oxidation of methionine. The default search parameters used were: enzyme, trypsin; max. missed cleavages, 1; fixed modifications, carbamidomethyl (C); variable modifications, oxidation (M); peptide tolerance, +0.4 Da; fragment mass tolerance +0.4 Da; protein mass, unrestricted; instrument = default [27].

### 2.9. Pathway Analysis

To determine which molecular pathways and diseases/functions were associated with the proteins that showed altered abundance, IPA (Ingenuity Pathway Analysis v. 2018) (www.ingenuity.com (accessed on 21 July 2020)) was used based on the corresponding human homolog of each protein in the Ingenuity Pathways Knowledge Base.

### 2.10. Statistical Analysis

All values were expressed as mean ± S.E.M. in “*n*” experiments. The data were expressed as percentage of maximum response to agonist and analyzed by interactive nonlinear regression through the computer program Graph Pad Prism version 8.0.0 (San Diego, CA, USA). E_max_ and –LogEC_50_/pD_2_ were calculated in Graph Pad Prism. The -LogEC_50_ and CRC shift were compared for each set of experiments. The GraphPad QuickCalcs “*t*” test (unpaired) was used to determine the level of significance and to analyze the data. A *p*-value < 0.05 was considered to be statistically significant.

## 3. Results

### 3.1. Isometric Contraction Study

#### Histamine- and KCl-Induced Concentration-Related Contractile Responses at pH_0_ 7.4, 6.8, and 6.0

**Figure 1:**
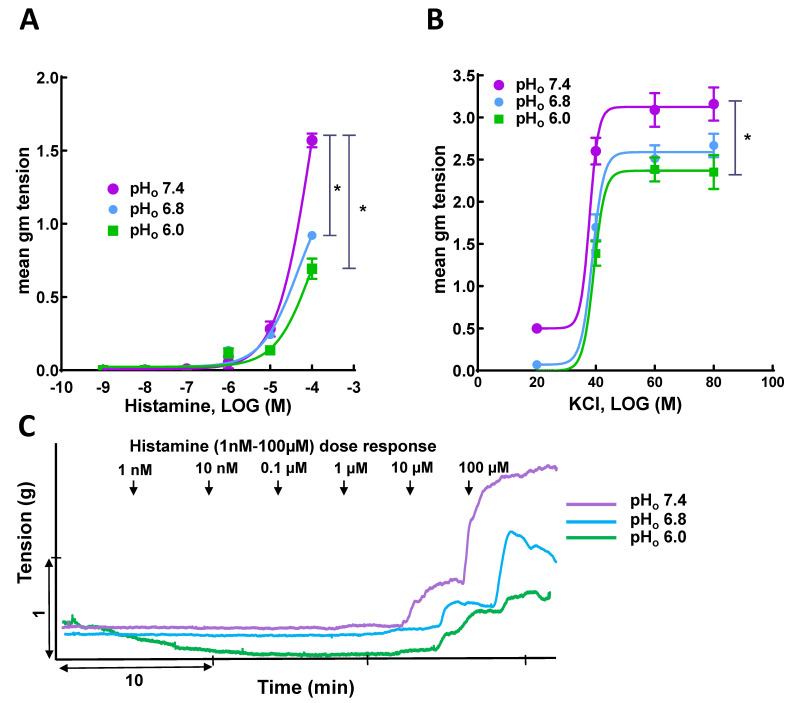
Effect of acidosis on agonist-induced contractile response in superior mesenteric artery (SMA): (**A**) Histamine-; (**B**) KCl-induced contractile responses in SMAs elicited at pH_o_ 7.4 are attenuated with a leftward shift of the CRC at pH_o_ 6.8 and 6.0; Representative tracing (**C**) showing histamine-induced contractile responses in SMA rings at pH_o_ 7.4, 6.8, and 6.0. * *p* < 0.05, comparison vs. normal pH_o_ (7.4), *n* = 6–14.

A previous study from our lab showed that acidosis attenuated NA- and PE-induced contractile responses due to reduced expression of α1D-adrenergic receptor and enhanced release of endothelium-relaxing factors in SMAs [19]. Hence, in this study, we aimed to determine if this attenuation effect of acidosis on vasocontractile machinery was specific to GPCR signaling. To discern the effect of acidosis on GPCR versus non-GPCR signaling, we employed histamine, a non-adrenergic GPCR agonist, and KCl (K^+^-depolarization) to elicit contraction at pH_o_ 7.4, 6.8, and 6.0 in SMA rings. Table 1 shows the histamine- and KCl-induced maximal contractile responses (E_max_) at each pH_o_. The histamine- and KCl-induced contractile response curves (CRCs) are presented in Figure 1A,B. The histamine-induced contractile response at pH_o_ 7.4 was attenuated at pH_o_ 6.8, with a shift of the CRC to the right, and this attenuation was even greater at pH_o_ 6.0, with a further shift of the CRC to the right.

The KCl-induced CRCs are presented in Figure 1B. The KCl-induced CRC at pH_o_ 7.4 was shifted to the right, with a significant decrease in E_max_ (*p* < 0.05) at pH_o_ 6.0. This suggests that acidosis attenuates the vascular contractile machinery as such and is independent of receptor stimulation.

### 3.2. Ach- and SNP-Induced Vasorelaxation in NA- and KCl-Precontracted SMA Rings

**Figure 2:**
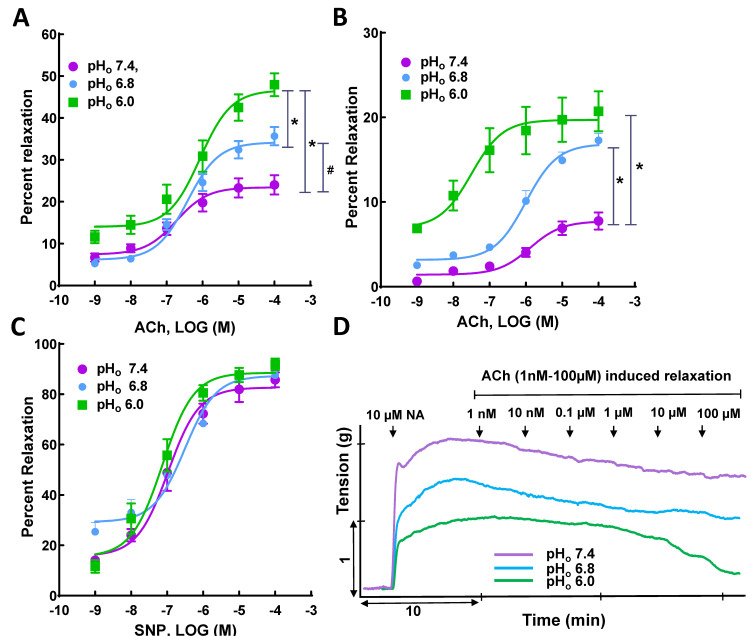
Effect of acidosis on vasorelaxation in superior mesenteric artery (SMA). Ach-induced vasorelaxation in (**A**) noradrenaline- and (**B**) KCl-precontracted SMA rings at pH_o_ 7.4 is augmented at acidic pH_o_ (6.8 and 6.0); (**C**) SNP-induced vasorelaxation in NA-precontracted SMA rings did not differ significantly at pH_o_ 7.4, 6.8, and 6.0; representative tracing showing (**D**) Ach-induced vasorelaxation in NA-precontracted SMA rings at pH_o_ 7.4, 6.8, and 6.0. This suggests endothelium plays a pivotal role in acidosis-mediated relaxation in SMA. * *p* < 0.05 vs. normal pH_o_ (7.4) and ^#^ *p <* 0.05 vs. mild acidic pH_o_ (6.8), *n* = 5–9.

A previous study from our lab showed that mildly acidic conditions (pH_o_ 6.8) augmented vasorelaxation due to an increased release of nitric oxide at basal state and that this effect was enhanced in Ach-induced relaxation [18]. In this study, we sought to extend our findings to investigate the effect of severe acidic conditions (pH_o_ 6.0) on vasorelaxation in SMAs. Table 2 shows the mean maximal vasorelaxation values for NA- and SNP-precontracted SMA rings in response to Ach and SNP (1 nM–100 µM) at pH_o_ 7.4, 6.8, and 6.0. Ach-induced vasorelaxation in NA-precontracted SMA rings at pH_o_ 7.4 was augmented, with a shift of the CRC to the left, at pH_o_ 6.8 and 6.0, along with a significant increase in E_max_ (approximately two-fold, at pH_o_ 6.0 *p* < 0.05) (Figure 2A). Similarly, in KCl-precontracted vessels, the Ach-induced vasorelaxation elicited at pH_o_ 7.4 was also increased, with a shift of CRC to left, at pH_o_ 6.8, together with a significant increase in E_max_ (approximately four-fold, *p* < 0.05), and at pH_o_ 6.0, with a significant increase in E_max_ (> four-fold, *p* < 0.05) (Figure 2B). The CRCs for SNP at pH_o_ 7.4 remained unaltered at pH_o_ 6.8 and 6.0 (Figure 2C). In conclusion, Ach-induced vasorelaxation in NA- and KCl-precontracted SMA rings was augmented by a decrease in pH_o_ from 7.4 to 6.0, whereas SNP-induced vasorelaxation did not differ significantly at pH_o_ 7.4, 6.8, and 6.0. This suggests acidosis augments endothelium-dependent vasorelaxation in SMAs.

### 3.3. Proteomic Analysis of Whole Mesenteric Artery Wall Extracts by 2D-GE and MS

**Figure 3:**
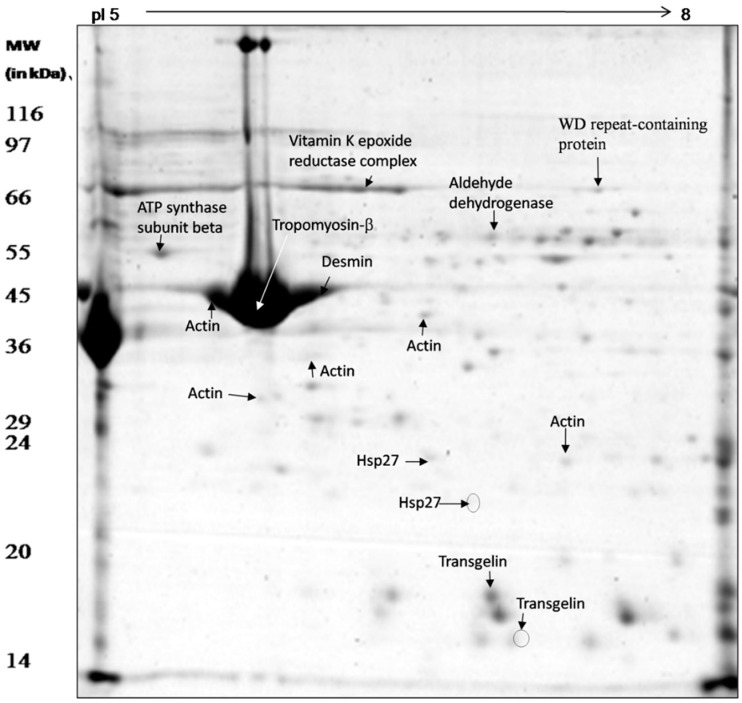
Proteome map of *C. hircus* mesenteric artery vessel wall proteins showing the protein spots identified by mass spectrometry (LC-MS/MS and MALDI TOF/MS).

Soluble SMA vessel proteins were separated into 120 discrete spots by 2D PAGE (Figure 3). The majority of the separated protein spots were found between pH 6–8. Unique protein spots revealing changes in abundance with respect to change in pH were isolated from the gel for identification by mass spectrometry (Figure 3). A total of sixteen spots were excised and characterized by LC-MS/MS and MALDI TOF/MS (Table 3 and Table 4). Five out of sixteen spots were identified as actin, out of which three (G13, G14, and G19) were more than two-fold more abundant under acid stress, while the other two (G10 and G26) did not show any change. Two proteins were identified as the actin-binding protein transgelin, of which one positional variant (or proteoform) was less than two-fold more abundant (G8), whereas the other did not show any change under low pH stress (G15). Two proteins were identified as Hsp27; one of these was found to be more abundant (G3) and another absent (G4) in the acid-stressed tissue. One protein spot, identified as ATP synthase (G23), was more than two-fold abundant under acid stress. One protein identified as WD repeat-containing protein 1 (G17) was five-fold more abundant in acid-stressed tissues. A protein identified as aldehyde dehydrogenase (G18) was more abundant (two-fold) and another identified as pyruvate kinase (G20) disappeared under acid stress (Table 3). Three proteins were identified by MALDI TOF/MS as tropomyosin (G12), desmin (G11) and vitamin K epoxide reductase complex (G16); tropomyosin showed no change in abundance, whereas the abundance of the other two proteins increased more than two- and three-fold, respectively, under acid stress (Table 4).

### 3.4. Pathway Analysis

**Figure 4:**
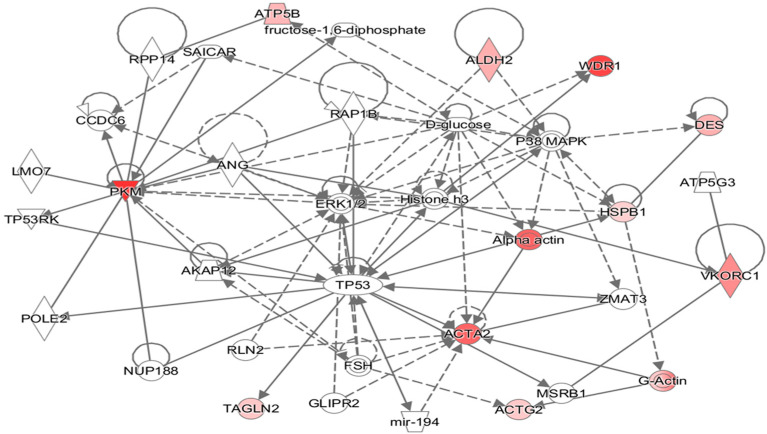
Putative biochemical networks affected by thermal exposure, as revealed by Ingenuity Pathway Analysis (IPA). Differentially abundant proteins showing a +/− 2-fold change or greater (*p* < 0.05) were selected and entered into the IPA Software to identify potential interactions. Solid arrows represent known physical interactions, while dotted arrows represent indirect interactions. Red shapes indicate proteins in this network that were identified by 2D-GE and mass spectrometric analysis. The proteins were mapped to their corresponding human homologues in the Ingenuity Pathways Knowledge Base. The homologues used in this study are as follows: ATP5B, ATP synthase subunit β mitochondrial; HSPB1, Hsp27; ACTA2, actin aortic smooth muscle; ACTG2, actin γ; TAGLN, transgelin; WDR1, WD repeat-containing protein; ALDH2, aldehyde dehydrogenase; PKM, pyruvate kinase; VKROC1, vitamin K epoxide reductase complex, subunit 1-like 1; DES, desmin.

All the differentially expressed proteins were mapped to their homologs and were assigned to biological functions in the IPA database. All the proteins were found to be involved in a single network (Figure 4). Cellular movement, cell death and survival, small biomolecules biochemistry, as well as carbohydrate and nucleic acid metabolism were found to be functions/diseases with the highest number of proteins involved. The highest numbers of proteins were involved in cellular growth and maintenance (six proteins), followed by cell death (five proteins), and cell movement (five proteins). The proteins affecting these processes are given in Figure S1 (Supplementary File S). The proteins aldehyde dehydrogenase (ALDH2), transgelin (TALGN2), pyruvate kinase (PKM), and Hsp27 were among those known to inhibit apoptosis, while the proteins vitamin K epoxide reductase complex (VKROC1), transgelin (TALGN2), pyruvate kinase (PKM), and Hsp27 were among those known to promote cell proliferation. Hsp27 is also known to be associated with inhibition of cerebrovascular dysfunction and actin (ACTG2) with suppressing relaxation of the heart and heart rate.

## 4. Discussion

In our isometric contraction study, we observed that low pH significantly attenuated the development of vascular tension in response to both histaminergic receptor H_1_-mediated and non-receptor, KCl-mediated contractile responses. Similarly, under acidosis, Ach-induced vasorelaxation was augmented, while SNP-induced vasorelaxation was not. We also observed that acetylcholine, at the lowest concentration tested (1 nM), relaxed SMA rings about 5% (25% of total relaxation in control) at pH_o_ 6.8 and about 10% (50% of the total relaxation in control), which suggests the vessel was losing tonus and that acidosis, per se, was inducing relaxation. A previous report from our lab also showed that acidosis itself induced vasorelaxation in SMAs, independent of acetylcholine stimulation, through nitric oxide (NO) signaling, as represented by upregulated basal NO levels under acidosis [18]. Hence, decreased pH_o_ had a common effect that speaks about the robust nature of this attenuated contractile response and augmented endothelium-dependent vasorelaxation in SMA rings. The signaling peptides responsible for the observed pharmacological events were investigated using proteomics tools, including 2D-GE and MS. A recent study showed that induction of hypercapnia through exposure to CO_2_ for 5 min was sufficient to induce cerebrovascular dilation through activation of acid-sensing ion channel-1A (ASIC1A), a proton-gated cation channel, resulting in cerebrovascular hypoperfusion [28]. The current study used an ex vivo model of acute acidosis, which involved incubating arterial rings for 90 min at acidic pH_o_, a standard experimental tool to study the effects of acidosis on the contractile response in isolated arterial rings [18,19,29,30]. Consequently, the findings from our study will help in understanding the pathophysiological alterations contributing to hypotension and hypovolemic shock during acidosis.

To date, proteomic aspects of vascular tissues, including superior mesenteric arteries, have received little attention, both under physiological and patho-physiological conditions. The present functional studies, with classical pharmacology combined with cutting-edge proteomics, is a modest attempt to connect the functional observations with the molecular signals involved in the microenvironment of the vessel wall. Considering that proteomes change in a highly dynamic manner under acid stress (change in pH in the tissue environment, either acidic or alkaline), we employed gel-based proteomics, i.e., 2D-GE followed by LC-MS/MS and/or MALDI TOF/MS, to investigate changes in soluble SMA vessel proteins (Table 3 and Table 4). It was noted that a high abundance of actin was observed in the soluble protein extract of SMAs (Table 3), which could be indicative of muscle protein degradation following pH stress. Metabolic acidosis often leads to loss of body protein mainly due to accelerated protein breakdown in muscle [31,32]. It has been shown that metabolic acidosis stimulated muscle protein degradation by activating an ATP-dependent pathway involving ubiquitin and proteasomes and that an increase in protein degradation in acidic muscles was eliminated when these muscles were depleted of ATP by treatment with inhibitors of respiration and glycolysis breakdown [32]. Interestingly, in our study, ATP synthase subunit β (mitochondrial) (ATPB) was found to be upregulated (Table 3), which may indicate the contribution of the ATP-dependent proteolytic process to the increased muscle protein degradation associated with metabolic acidosis [32]. Moreover, a previous study suggested that increased lysine acetylation of mitochondrial ATP synthase subunit β may play a role in the response of the proximal convoluted tubules to chronic metabolic acidosis [33]. Oxygen availability is a key limiting factor in acid stress, and the viability of cells with high energy demands relies on the continuous supply of ATP for successful muscle contraction-relaxation cycles. It was recently found that mild extracellular acidosis was a physiological consequence of anaerobic metabolism and reprogramed mitochondrial metabolism to preserve efficient ATP production, regardless of oxygen levels [34].

Previously, proteomic profiling of rat renal proximal convoluted tubules has shown differential abundance of cytosolic proteins, including ATP synthase, actin and transgelin (TAGLN), following metabolic acidosis [1]. In this study, transgelin was also found to be upregulated in the acid-stressed condition (Table 3). Two more proteins identified by MALDI TOF/MS are associated with the muscle contraction mechanism, desmin and tropomyosin, indicating an effect of acidosis on the skeletal-muscular system.

WD repeat-containing protein 1 (WDR1) has been found to induce disassembly of actin filaments [1] and was found to be upregulated in this study. This corroborates with a similar finding of Schauer et al., where this protein was found to be upregulated more than three-fold in the rat renal proximal convoluted tubules under the influence of metabolic acid stress [1].

The heat shock proteins (hsp) are known to function as molecular chaperones. In addition to their role in de novo protein folding, hsps are involved in various aspects of proteome maintenance, including macromolecular-complex assembly, protein transport and degradation, as well as well as aggregate dissociation and refolding of stress-denatured proteins [35]. Various stress conditions induce a regulated response resulting in increased expression of some HSPs, which helps to maintain protein homeostasis. The alteration in expression of Hsp27 following acid stress observed in this study indicates its association with acidosis [36]. It has been reported earlier that, in addition to its chaperoning activities, heat shock proteins protect cultured astrocytes against acidosis [37]. Hence, it could be predicted that Hsp27 expression may be augmented with increases in acidosis in vascular muscle cells to buffer against acidosis-induced cellular alterations.

In a previous study, the activities of several glycolytic enzymes, including pyruvate kinase, were reported to be increased under the influence of metabolic acidosis in rat submandibular glands [38]. In the present study, it was found that pyruvate kinase (PK) levels also increased in SMAs following acid stress.

Vitamin K epoxide reductase complex subunit 1 (VKORC1L1) is responsible for reducing vitamin K 2,3-epoxide to convert it to the enzymatically activated form, which is essential for blood clotting. This enzyme was found to be increased more than three-fold following acid stress (Table 4). It has been reported earlier, in a case study, that treatment of vitamin K deficiency led to a complete recovery from severe lactic acidosis in a 3-year-old boy [39]. Thus, this protein may have some curative association with acidosis, which needs to be further studied.

Aldehyde dehydrogenase (ALDH) was found to be increased in abundance in the proteome of SMAs under acidic stress, which corroborates with a previous case report where this protein was found to be associated with lactic acidosis [40].

The attenuation of vasocontraction and augmentation of vasorelaxation observed under acid stress in SMA rings might have evoked a series of translational protein expression changes, including signaling molecules, which we have investigated using a proteomics platform. In this study, alterations were observed in the abundance of actin proteoforms and other actin-associated proteins. Proteoforms are variations in proteins arising from a single gene via multiple mechanisms, such as alternative splicing and post-translation modifications [41]. The proteoforms vary in their expression patterns in physiological states, and thus, have been used as biomarkers of health and diseases [42]. The variations observed in the proteoforms of actin could be indicative of impairment in muscular contractility under acid stress and could have importance as biomarkers and need further investigation. Similarly, the increase in ATP synthase β could be due to mitochondrial metabolic pathway readjustment to preserve efficient ATP production for successful muscle contraction-relaxation cycle. Together, these results suggest new insights into a possible adaptive mechanism for maintenance of vascular tone under acute acid stress through upregulation of actin proteoforms, actin-associated proteins, and mitochondrial ATP synthase.

We are aware of some methodological limitations of our study. In particular, the use of 7-cm IPG strips and of mini-gels for the two-dimensional gel electrophoretic separation of total artery wall tissue extracts may have limited the identification of other key proteins, as it might not allow sufficient discrimination for the expected complexity of total tissue extracts and sufficient quantitative accuracy in the evaluation of proteins differentially expressed between control ant test samples and in the comparisons between replicate gels.

Some of the technical limitations in this study can be overcome in future work by the use of longer IPG strips with extended pH gradients, larger 2D gels, and improved labeling techniques, such as two-dimensional difference gel electrophoresis (2D-DIGE). However, our goal was to identify key molecules most prominently associated with acid stress-induced vasorelaxation as prospective candidates for future studies. Further investigation is also warranted, using non-gel-based proteomic techniques for a deeper understanding of the vascular changes associated with therapeutic regimens [43].

## Figures and Tables

**Table 1:** Histamine- (1 nM–100 µM) and KCl (20–80 mM)-induced contractile response at pH_o_ 7.4, 6.8, and 6.0 in SMA rings. Data were expressed as mean gram tension ± SE. *n* = no of experiments.

		pH_o_ 7.4	pH_o_ 6.8	pH_o_ 6.0
Histamine	E_max_	1.57 ± 0.05 (*n* = 12)	0.92 ± 0.02 * (*n* = 6)	0.69 ± 0.07 * (*n* = 14)
KCl	E_max_	3.16 ± 0.20 (*n* = 7)	2.67 ± 0.14 (*n* = 10)	2.35 ± 0.20 * (*n* = 7)

* *p* < 0.05 versus pH 7.4 (data were compared between sub-rows, within columns under E_max_ and pD_2_).

**Table 2:** Ach- or SNP (1 nM–100 µM)-induced vasorelaxation on NA- (10 µM) or KCl (60 mM)-precontracted SMA rings at pH_o_ 7.4, 6.8, and 6.0. The data presented as mean ± SE. *n* = no of experiments.

		E_max_/E_Bmax_ (%)			−Log EC_50_ (pD_2_)		
		pH_o_ 7.4	pH_o_ 6.8	pH_o_ 6.0	pH_o_ 7.4	pH_o_ 6.8	pH_o_ 6.0
NA	Ach	24.01 ± 2.31 (*n* = 19)	35.66 ± 2.18 * (*n* = 13)	47.94 ± 2.73 * (*n* = 19)	6.73 ± 0.24	6.40 ± 0.18	6.01 ± 0.37 *^#^
	SNP	85.75 ± 2.98 (*n* = 8)	87.57 ± 2.32 (*n* = 8)	91.91 ± 2.31 (*n* = 6)	6.98 ± 0.13	6.51 ± 0.17	7.13 ± 0.13 *^#^
KCl	Ach	4.06 ± 0.71 (*n* = 4)	16.10 ± 2.67 * (*n* = 4)	17.33 ± 2.97 * (*n* = 7)	5.91 ± 0.26	6.04 ± 0.23	7.12 ± 0.44 *^#^

* *p* < 0.05 versus pH 7.4, ^#^ *p* < 0.05 versus pH 7.4 (data were compared between subcolumns, within rows under E_max_ and pD_2_).

**Table 3:** Protein spots identified by LC-MS/MS in mesenteric artery proteomes. *↑* indicates upregulation of the protein spot. Fold change value was calculated by using the 2D gel image analysis software PD Quest (Bio-Rad) version 7.20. A value of *p* < 0.05 was considered to be statistically significant.

Spot Name	Protein ID	Protein Name	Species	Protein Mass	Coverage	Isoelectric Point	# of Unique Peptides	# of Unique Spectrums	Fold Change	*p*-Value
G3	gi|389620461|gb| AFK93550.1|	Hsp27 proteinpartial	*C. hircus*	20,543.41	62.84	6.68	11	85	1.41 ↑	0.001
G4	gi|389620461|gb| AFK93550.1|	Hsp27 proteinpartial	*C. hircus*	20,543.41	62.3	6.68	13	81	Absent in exp	0.001
G23	gi|548466133|ref|XP_005680388.1|	Predicted: ATP synthase subunit β mitochondrial	*C. hircus*	56,148.48	80.87	4.92	34	537	2.14 *↑*	0.005
G13	gi|548522357|ref|XP_005698249.1|	Predicted: actinaortic smooth muscle	*C. hircus*	42,380.96	48.81	5.05	8	73	4.53 ↑	0.004
G14	gi|548485020|ref|XP_005686409.1|	Predicted: actin γ-enteric smooth muscle isoform X1	*C. hircus*	42,248.94	78.72	5.16	1	1	1.61 ↑	0.002
G19	gi|548522357|ref|XP_005698249.1|	Predicted: actin aortic smooth muscle	*C. hircus*	42,380.96	70.82	5.05	5	49	2.99 *↑*	0.001
G10	gi|548522357|ref|XP_005698249.1|	Predicted: actin aortic smooth muscle	*C. hircus*	42,380.96	87.8	5.05	1	14	No change	0.38
G26	gi|548485020|ref|XP_005686409.1|	Predicted: actin γ-enteric smooth muscle isoform X1	*C. hircus*	42,248.94	89.89	5.16	1	1	No change	0.21
G8	gi|548494696|ref|XP_005689548.1|	Predicted: transgelin	*C. hircus*	22,609.45	78.61	9.29	22	285	1.67 ↑	0.002
G15	gi|548494696|ref|XP_005689548.1|	Predicted: transgelin	*C. hircus*	22,609.45	65.67	9.29	14	102	Absent in exp	0.001
G17	gi|548471260|ref|XP_005682023.1|	Predicted: WD repeat-containing protein 1(partial)	*C. hircus*	65,402.72	47.55	6.47	22	230	5.4 *↑*	0.001
G18	gi|548501256|ref|XP_005691616.1|	Predicted: aldehyde dehydrogenase mitochondrial	*C. hircus*	46,336.44	56.4	6.08	15	120	2.39 *↑*	0.003
G20	gi|548481183|ref|XP_005685233.1|	Predicted: pyruvate kinase isoform X1	*C. hircus*	58,513.25	36.16	7.76	17	58	Absent in cont	0.001

**Table 4:** Protein spots identified by MALDI TOF/MS of mesenteric artery proteomes. *↑* indicates upregulation of the protein spot. Fold change value was calculated by using the 2D gel image analysis software PDQuest (Bio-Rad) version 7.20. A value of *p* < 0.05 was considered to be statistically significant.

Spot Name	Protein ID	Protein Mass (Daltons)	Protein Score	pI	% Sequence Coverage	Peptide Searched/Matched	Proteins Identified	Species	Fold Change	*p*-Value
G16	gi|358420988|ref|XP_003584789.1|	17,854.71	51.2	9.3	17.31	27/156	Vitamin K epoxide reductase complex, subunit 1-like 1	*B. taurus*	3.38 ↑	0.001
G11	gi|28189827|dbj|BAC56528.1|	215,154.23	323	4.49	46.70	85/182	Desmin	*B. taurus*	2.34 ↑	0.002
G12	gi|87196510|ref|NP_001010995.2|	33,048.59	219	4.34	32.04	91/284	Tropomyosin-β chain	*B. taurus*	No change	0.27

## Data Availability

Data is contained within the article and Supplementary Materials.

## Supplementary Materials

The following supporting information can be downloaded at: https://www.mdpi.com/article/10.3390/genes13050801/s1, Figure S1: Proteins involved in the major functions/diseases (A) apoptosis (B) proliferation of cells (C) cerebrovascular dysfunction, relaxation of heart and (D) cell movement occurring during acid stress as revealed by IPA analysis. The proteins aldehyde dehydrogenase (ALDH2), transgelin (TALGN2), pyruvate kinase (PKM), and Hsp27 were found to be inhibiting the apoptosis process while proteins, Vitamin K epoxide reductase complex (VKROC1), transgelin (TALGN2), pyruvate kinase (PKM), and Hsp27 were found to be activating the proliferation of cells. Hsp27 is reported to be associated with inhibition of cerebrovascular dysfunction and actin (ACTG2) with inhibition of relaxation of heart and heart rate.

## Abbreviations

Ach, acetylcholine; [Ca^2+^]_i_, intracellular Ca^2+^; 2D-PM, two-dimensional peptide mapping; 2D-GE, two-dimensional polyacrylamide gel electrophoresis; ACTA2; actin aortic smooth muscle; ACTG2, actin γ; ALDH2, aldehyde dehydrogenase; ATP5B, ATP synthase subunit β mitochondrial; CBB, Coomassie Brilliant Blue; CRCs, concentration response curves; DES, desmin; eNOS, endothelial nitric oxide synthetase; EC, endothelial cell; EC_50,_ half maximal effective concentration; E_max_, maximum response/effect maximum; IEF, isoelectrofocussing; KCl, potassium chloride; LC-MS/MS, liquid chromatography- mass spectrometry; MALDI TOF/MS, matrix-assisted laser desorption/ionization time-of-flight mass spectrometry; MKHS, modified Krebs-Henseleit solution; NA, L- noradrenaline; NO, nitric oxide; PAGE, Polyacrylamide gel electrophoresis; pD2, negative logarithm of an EC50; PE, phenylephrine hydrochloride; pH_o_, extracellular pH; pH_i_, intracellular pH; SMA, superior mesenteric artery; SNP, sodium nitroprusside; TAGLN, transgelin; WDR1, WD repeat-containing protein; PKM, pyruvate kinase; VKROC1, vitamin K epoxide reductase complex, subunit 1-like 1.

## References

[1] Schauer K.L., Freund D.M., Prenni J.E., Curthoys N.P. (2013). Proteomic profiling and pathway analysis of the response of rat renal proximal convoluted tubules to metabolic acidosis. Am. J. Physiol.-Ren. Physiol..

[2] Takeuchi N. (2012). Non-Occlusive Mesenteric Ischemia during the Course of Heart Failure. J. Clin. Case Rep..

[3] Lim S.Y., Park Y., Chin H.J., Na K.Y., Chae D.-W., Kim S. (2019). Short-term and long-term effects of low serum bicarbonate level at admission in hospitalised patients. Sci. Rep..

[4] Căpuşă C., Ştefan G., Stancu S., Lipan M., Tsur L.D., Mircescu G. (2017). Metabolic acidosis of chronic kidney disease and subclinical cardiovascular disease markers: Friend or foe?. Medicine.

[5] Kraut J.A., Madias N.E. (2010). Metabolic acidosis: Pathophysiology, diagnosis and management. Nat. Rev. Nephrol..

[6] Caliskan A., Yavuz C., Karahan O., Demirtas S., Yazici S., Guclu O., Mavitas B. (2014). Serum ischaemia–modified albumin level is an irrelevant predictive factor for ischaemic duration in mesenteric ischaemia. Perfusion.

[7] Gokina N.I., Fairchild R.I., Bishop N.M., Dawson T.E., Prakash K., Bonney E.A. (2021). Kinetics of Postpartum Mesenteric Artery Structure and Function Relative to Pregnancy and Lactation in Mice. Reprod. Sci..

[8] Watters A., Gibson D., Dee E., Mascolo M., Mehler P.S. (2020). Superior mesenteric artery syndrome in severe anorexia nervosa: A case series. Clin. Case Rep..

[9] Liang J., Xing H., Chang Y. (2019). Thermal damage width and hemostatic effect of bipolar electrocoagulation, LigaSure, and Ultracision techniques on goat mesenteric vessels and optimal power for bipolar electrocoagulation. BMC Surg..

[10] Alvites R.D., Branquinho M.V., Sousa A.C., Lopes B., Sousa P., Mendonça C., Atayde L.M., Maurício A.C. (2021). Small Ruminants and Its Use in Regenerative Medicine: Recent Works and Future Perspectives. Biology.

[11] Wu X., Han X., Li L., Fan S., Zhuang P., Yang Z., Zhang Y. (2020). iTRAQ-based quantitative proteomics and target-fishing strategies reveal molecular signatures on vasodilation of Compound Danshen Dripping Pills. Chem.-Biol. Interact..

[12] Rodrigues T., Bernardes G.J.L. (2020). Machine learning for target discovery in drug development. Curr. Opin. Chem. Biol..

[13] Saviola A.J., Negrão F., Yates J.R. (2020). Proteomics of Select Neglected Tropical Diseases. Annu. Rev. Anal. Chem..

[14] McCall M.N., Kent O.A., Yu J., Fox-Talbot K., Zaiman A.L., Halushka M.K. (2011). MicroRNA profiling of diverse endothelial cell types. BMC Med. Genom..

[15] Bianchi L., Lorenzoni P., Bini L., Weber E., Tani C., Rossi A., Agliano M., Pallini V., Sacchi G. (2007). Protein expression profiles ofBos taurus blood and lymphatic vessel endothelial cells. Proteomics.

[16] Walmsley S.J., Freund D.M., Curthoys N.P. (2012). Proteomic profiling of the effect of metabolic acidosis on the apical membrane of the proximal convoluted tubule. Am. J. Physiol.-Ren. Physiol..

[17] Nakanishi T., Gu H., Momma K. (1997). Developmental changes in the effect of acidosis on contraction, intracellular pH, and calcium in the rabbit mesenteric small artery. Pediatr. Res..

[18] Mohanty I., Parija S.C., Suklabaidya S., Rattan S. (2018). Acidosis potentiates endothelium-dependent vasorelaxation and gap junction communication in the superior mesenteric artery. Eur. J. Pharmacol..

[19] Mohanty I., Suklabaidya S., Parija S.C. (2016). Acidosis reduces the function and expression of α_1D_ -adrenoceptor in superior mesenteric artery of *Capra hircus*. Indian J. Pharmacol..

[20] Parija S.C., Mohanty I. (2018). Acid stress reduces the function of Na^+^-K^+^ATPase in superior mesenteric artery of *Capra hircus*. Pharmacogn. Mag..

[21] Bhattacharjee S., Mohanty S., Sharma A.P., Mohanty B.P. (2011). Effect of storage temperature as a preanalytical variable on the lens crystallins protein quality for proteomic studies. Proteom. Clin. Appl..

[22] Bradford M.M. (1976). A rapid and sensitive method for the quantitation of microgram quantities of protein utilizing the principle of protein-dye binding. Anal. Biochem..

[23] Laemmli U.K. (1970). Cleavage of Structural Proteins during the Assembly of the Head of Bacteriophage T4. Nature.

[24] O’Farrell P. (1975). High resolution two-dimensional electrophoresis of proteins. J. Biol. Chem..

[25] Martin S.A.M., Mohanty B.P., Cash P., Houlihan D.F., Secombes C.J. (2007). Proteome analysis of the Atlantic salmon (Salmo salar) cell line SHK-1 following recombinant IFN-γ stimulation. Proteomics.

[26] Bringans S., Eriksen S., Kendrick T., Gopalakrishnakone P., Livk A., Lock R., Lipscombe R. (2008). Proteomic analysis of the venom ofHeterometrus longimanus (Asian black scorpion). Proteomics.

[27] Mahanty A., Purohit G.K., Banerjee S., Karunakaran D., Mohanty S., Mohanty B.P. (2016). Proteomic changes in the liver of *Channa striatus* in response to high temperature stress: Proteomics and 2-DE. Electrophoresis.

[28] Stark R.J., Choi H., Lamb F.S. (2019). Neuronal ASIC1A as a Cerebral pH Sensor. Circ. Res..

[29] Capellini V.K., Restini C.B.A., Bendhack L.M., Evora P.R.B., Celotto A.C. (2013). The Effect of Extracellular pH Changes on Intracellular pH and Nitric Oxide Concentration in Endothelial and Smooth Muscle Cells from Rat Aorta. PLoS ONE.

[30] Yartsev V.N., Karachentseva O.V. (2017). Noradrenaline-Induced Restoration of Acidosis-Inhibited Neurogenic Vasoreactivity at Using Different Electrical Stimulation Frequencies. Neurosci. Behav. Physiol..

[31] Workeneh B.T., Mitch W.E. (2010). Review of muscle wasting associated with chronic kidney disease. Am. J. Clin. Nutr..

[32] Mitch W.E., Medina R., Grieber S., May R.C., England B.K., Price S.R., Bailey J.L., Goldberg A.L. (1994). Metabolic acidosis stimulates muscle protein degradation by activating the adenosine triphosphate-dependent pathway involving ubiquitin and proteasomes. J. Clin. Investig..

[33] Freund D.M., Prenni J.E., Curthoys N.P. (2013). Response of the mitochondrial proteome of rat renal proximal convoluted tubules to chronic metabolic acidosis. Am. J. Physiol.-Ren. Physiol..

[34] Khacho M., Tarabay M., Patten D., Khacho P., MacLaurin J.G., Guadagno J., Bergeron R., Cregan S.P., Harper M.-E., Park D.S. (2014). Acidosis overrides oxygen deprivation to maintain mitochondrial function and cell survival. Nat. Commun..

[35] Kakkar V., Meister-Broekema M., Minoia M., Carra S., Kampinga H.H. (2014). Barcoding heat shock proteins to human diseases: Looking beyond the heat shock response. Dis. Models Mech..

[36] Singh A.K., Manns M.P., Seidler U. (2011). Cytoprotective effects of acidosis via heat shock protein HSP27 against the anticancer drug doxorubicin. Cell. Mol. Life Sci..

[37] Narasimhan P., Swanson R.A., Sagar S.M., Sharp F.R. (1996). Astrocyte survival and HSP70 heat shock protein induction following heat shock and acidosis. Glia.

[38] Bardoń A., Ceder O., Roomans G.M., Kollberg H. (1985). Effect of metabolic acidosis on glycolysis in rat submandibular glands. Res. Commun. Chem. Pathol. Pharmacol..

[39] Oriot D., Wood C., Gottesman R., Huault G. (1991). Severe Lactic Acidosis Related to Acute Thiamine Deficiency. J. Parenter. Enter. Nutr..

[40] Zosel A., Egelhoff E., Heard K. (2010). Severe Lactic Acidosis after an Iatrogenic Propylene Glycol Overdose. Pharmacotherapy.

[41] Carbonara K., Andonovski M., Coorssen J.R. (2021). Proteomes Are of Proteoforms: Embracing the Complexity. Proteomes.

[42] Forgrave L.M., Wang M., Yang D., DeMarco M.L. (2021). Proteoforms and their expanding role in laboratory medicine. Pract. Lab. Med..

[43] Mohanty B.P., Mahanty A., Mitra T., Mohanty S., Naik A.K., Parija S.C. (2020). Proteomic and transcriptomic changes in rat liver following oral feeding of formaldehyde. Chemosphere.

