# Supplementary figures and images for: Proteomic Profiling and Pathway Analysis of Acid Stress-Induced Vasorelaxation of Mesenteric Arteries In Vitro
Source: Genes (Basel). 2022 Apr 29;13(5):801. doi: 10.3390/genes13050801 (PMC9140505; doi:10.3390/genes13050801)

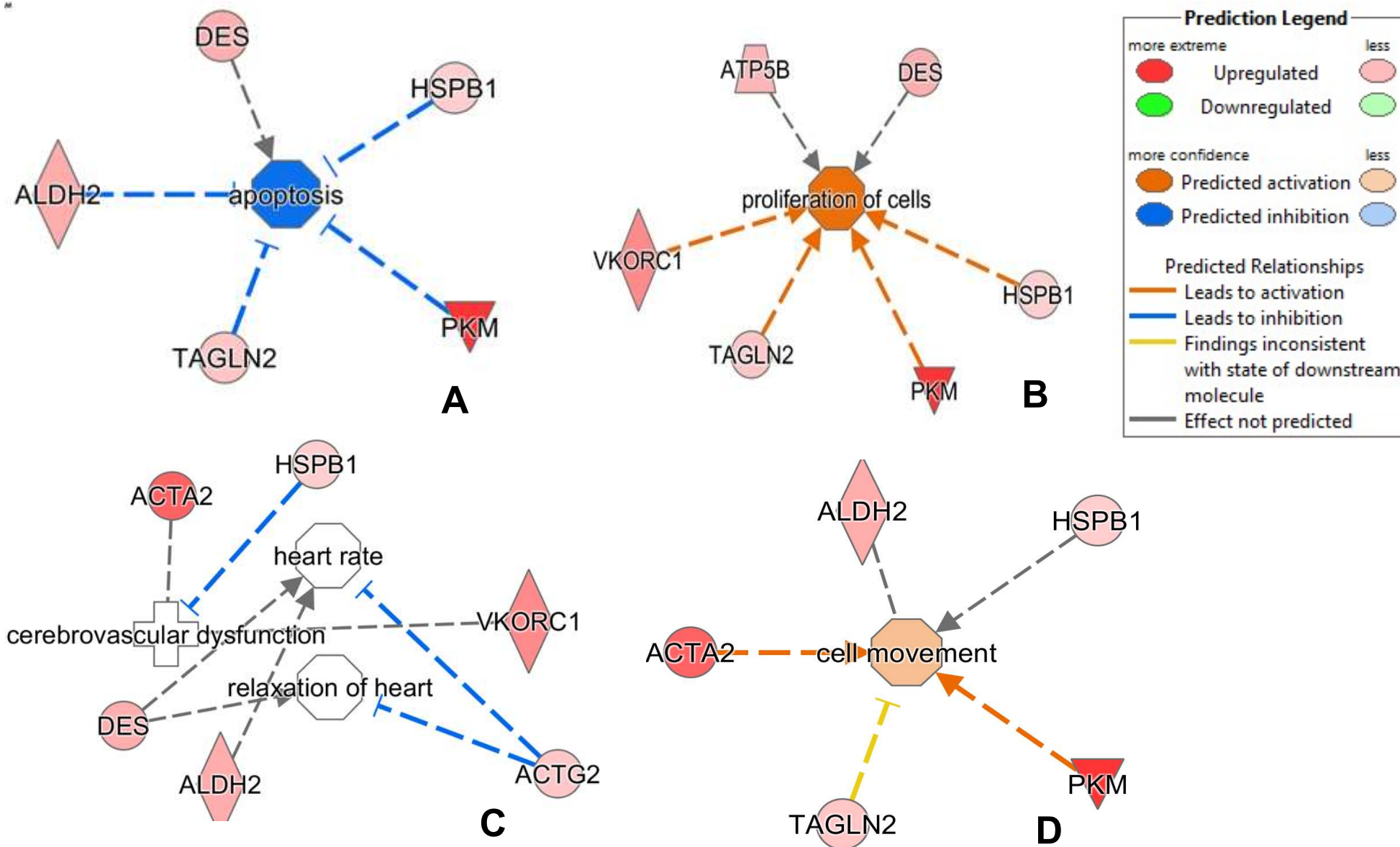

Supplement: Supplementary file 1 [file genes-13-00801-s001.zip › genes-1684783 Supplementary Figure S1.pdf]
